# Oral language intervention in the late primary school years is effective: evidence from a randomised control trial

**DOI:** 10.1111/jcpp.14084

**Published:** 2024-12-01

**Authors:** Rosanne Esposito, Arne Lervag, Charles Hulme

**Affiliations:** ^1^ University College London London UK; ^2^ University of Oxford Oxford UK; ^3^ University of Oslo Oslo Norway; ^4^ Oxford Brookes University Oxford UK

**Keywords:** Language, RCT, education, primary school intervention

## Abstract

**Background:**

Oral language skills provide the foundation for formal education, and children may require language support over an extended period of time to maximise their education potential. Most work on language intervention, however, has focussed on the preschool or early school years. Here, we describe the development and evaluation of the Oral Language for Literacy Intervention (OLLI) programme which is designed to support children with weak language skills in the later primary school years.

**Methods:**

We conducted a randomised control trial in 33 schools (50 classrooms). The language skills of all 8–9 year‐old children in each participating classroom (*n* = 1,423) were assessed using an automated app (LanguageScreen). The six children with the weakest LanguageScreen scores within each classroom (*n* = 296) were randomly allocated to the intervention (*n* = 148) or control group (*n* = 148). The children in the intervention group received the OLLI programme delivered in individual and small group sessions over 20 weeks. Children in the control group received their typical teaching.

**Results:**

Children receiving the OLLI programme made significantly larger gains than children in the control group on a preregistered latent variable reflecting standardised measures of oral language ability (*d* = 0.38) and on a measure of their written expression (*d* = 0.42).

**Conclusions:**

These findings have important implications for improving educational attainment in children in the late primary school years. The OLLI programme is designed to be deliverable at scale and is of relatively low cost.

## Introduction

Language skills are critical for many aspects of educational attainment including literacy (Hjetland, Brinchmann, Scherer, Hulme, & Melby‐Lervåg, 2020; Hulme, Nash, Gooch, Lervåg, & Snowling, 2015; Snow, 2016) and numeracy (Chow & Ekholm, 2019; Hornburg, Schmitt, & Purpura, 2018) as well as psycho‐social development (Norbury et al., 2016; van Agt, Verhoeven, van den Brink, & de Koning, 2011). Furthermore, it is well established that children from disadvantaged homes typically show poorer language development than children from more privileged backgrounds (Guo & Harris, 2000; Hart & Risley, 1995; Roulstone, Law, Rush, Clegg, & Peters, 2011; Sampson, Sharkey, & Raudenbush, 2008; Sirin, 2005). Language skills are, therefore, potentially a powerful contributor to the intergenerational transmission of social disadvantage: if children from disadvantaged homes enter school with poorer language skills, these may, in turn, adversely affect their educational attainments and reduce their occupational success. Interventions to improve language skills are, therefore, potentially a powerful way to reduce educational and occupational inequalities and may have positive effects on psychosocial development and well‐being.

There is now extensive evidence that school‐based interventions to improve language skills can be effective. Rogde, Hagen, Melby‐Lervåg, and Lervåg (2019) reported a meta‐analysis of 28 randomised controlled trials (RCTs) and 15 quasi‐experiments evaluating language interventions delivered either to whole classes or to small groups. The studies differed markedly in quality but overall, there was a small but significant effect of language intervention on oral language skills (*d* = 0.16) and studies with interventions to small groups demonstrated larger effects than whole‐class or larger group interventions (*d* = 0.25 vs. 0.10, respectively). Rogde et al. (2019) concluded that language interventions of good quality, especially when delivered to small groups, can have meaningful beneficial effects. These conclusions have been reinforced by later studies of the Nuffield Early Language Intervention (NELI) programme. West et al. (2021) reported a large‐scale cluster randomised trial evaluating the NELI programme in 193 schools. Children receiving the intervention showed greater gains in language skills than controls (*d* = 0.26 and 0.32 on two different latent variables measuring language ability). As a result of this study, the UK's Department for Education funded the delivery of NELI to many thousands of schools in England commencing in 2019. The effects of the NELI programme in this rollout were evaluated in an independent study using a Regression Discontinuity Design (https://educationendowmentfoundation.org.uk/projects‐and‐evaluation/projects/nuffield‐early‐language‐intervention‐scale‐up‐impact‐evaluation). This revealed that the NELI programme, when delivered at scale, produced effects that were similar in size to those obtained in the West et al. (2021) randomised trial.

In a subsequent study, West et al. (2024) reported the results of a cluster randomised trial evaluating a preschool language programme (the Nuffield Early Language Intervention – Preschool (NELI Preschool)). This programme consisted of a whole‐class language enrichment programme delivered to all children in preschool classrooms, coupled with targeted individual and small‐group language support delivered to the 5 children in each class with the weakest language skills. Children receiving NELI Preschool made significantly larger gains than the children in control group settings on a preregistered latent variable reflecting measures of oral language ability (enrichment children *d* = 0.26; enrichment + targeted children *d* = 0.16).

These previous studies clearly establish that language interventions in the preschool and early school years can be effective and lead to educationally meaningful improvements in language skills, and in some studies also lead to improvements in both single‐word reading (West et al. 2021) and reading comprehension skills (Fricke, Bowyer‐Crane, Haley, Hulme, & Snowling, 2013). The vast majority of this evidence, however, comes from studies of children between the ages of 3.5 and 5.5 years of age. Furthermore, it is clear that although such interventions are effective they are typically of relatively short duration (the NELI programmes are delivered over 2 school terms) and many children experience language weaknesses that are persistent. It is important therefore to provide evidence that language interventions for older children can also be effective. This is the focus of the present paper.

The present study builds upon an earlier study of language intervention for 8–9 year‐old children (Clarke, Snowling, Truelove, & Hulme, 2010). That study focussed on children with reading comprehension impairments and demonstrated that an oral language intervention delivered in three 30 min sessions per week (two individual and one dyadic session per week) was effective in improving children's vocabulary and reading comprehension skills. The study assessed vocabulary skills but did not include any broader measures of language comprehension and production. The current programme is based on the structure of the Oral Language programme developed by Clarke et al., but with completely new materials. The 20‐week OLLI programme is manualised and consists of two group sessions with three children per group and one individual session per week. In each session, the teaching assistant reads aloud a short piece of fiction, nonfiction or poetry around which the session activities are based. Children are taught definitions of targeted (Tier 2) vocabulary items, and these words are also embedded in the passages read to them. Interactive activities and games are used to support and reinforce learning. Children are taught to use the metacognitive strategies derived from reciprocal teaching of clarification, summarisation, prediction and questioning to improve their of understanding spoken language. Children are exposed to structured narratives which help to develop their own oral narrative productions during the programme. The OLLI programme shows some similarities to the Story Champs programme (Petersen, Staskowski, Spencer, Brough, & Foster, 2021), although there are major differences. Story Champs focuses primarily on whole class instruction for young children (kindergarten to Grade 3 in the United States) and explicitly teaches story grammar and vocabulary using fiction passages only. The OLLI programme focuses on older children and involves a wider range of passages (fiction, nonfiction and poetry), and a wider range of activities.

This study reports a randomised trial to assess the efficacy of the OLLI language intervention programme in 9‐year‐old children. We expected the OLLI programme to produce meaningful improvements in children's oral language and written expression. The trial was preregistered (https://www.isrctn.com/ISRCTN78621766). We use a latent variable assessing improvements on a range of standardised measures of language ability as our primary outcome. In addition, we use a measure of written expression that taps the ideas, vocabulary, sentence structure and grammar used in children's free writing. Free writing is a key educational outcome and one which might be expected to improve because of improvements in language skills. As a control measure, to assess whether there were general benefits from additional attention from teaching staff, we included arithmetic, which was not expected to improve because of the intervention.

## Method

An RCT was conducted in Year 4 classes in primary schools in England. Children in participating classes were randomly allocated to an intervention or waiting control group. Only children assigned to the treatment arm of the trial received the intervention, whilst children in the control group received their normal classroom instruction. The language and writing skills of the children in both arms of the trial were assessed before and after the intervention. The study design, assessment measures and analysis protocol were preregistered (https://www.isrctn.com/ISRCTN78621766). To avoid bias, the preregistered analyses were completed independently, blind to treatment allocation (by the second author, AL).

### Participants

In total, 62 Year 4 classes in 38 schools agreed to participate in the trial. All children in these classes were eligible to participate in the trial. Prior to commencing the initial screening of the children's language skills, 2 schools (containing 4 classes) withdrew. Therefore, initial language screening was conducted in 58 classes in 36 schools. In total, 1,423 children (boys *n* = 692 and girls *n* = 731) were screened. Of these, 565 children (40% of the sample) were registered as having English as an additional language (EAL). The 6 children with the weakest LanguageScreen scores within each classroom (*n* = 296) were randomly allocated to the intervention (*n* = 148) or control group (*n* = 148), minmising for LanguageScreen and Age using the randomise module in Stata 16.

### Design

School staff initially assessed all participating children using the LanguageScreen App (https://oxedandassessment.com/languagescreen/; t0) to identify the 6 children in each class with the weakest language skills. Following this screening, 8 classes in 5 schools withdrew from the trial due to Covid‐related staffing issues. The 6 children with the weakest language in each of the 50 remaining classes were then individually tested by the research team on a battery of standardised language assessments (t1). At t1 these 6 children also undertook a 15 min writing assessment and 2 min arithmetic test, administered by school staff.

Once all pretesting was complete, all teaching assistants deployed by their schools to deliver OLLI, together with a senior leader from each school, attended 2 half‐days of online training. The intervention was delivered between January and July 2022.

**Figure 1:**
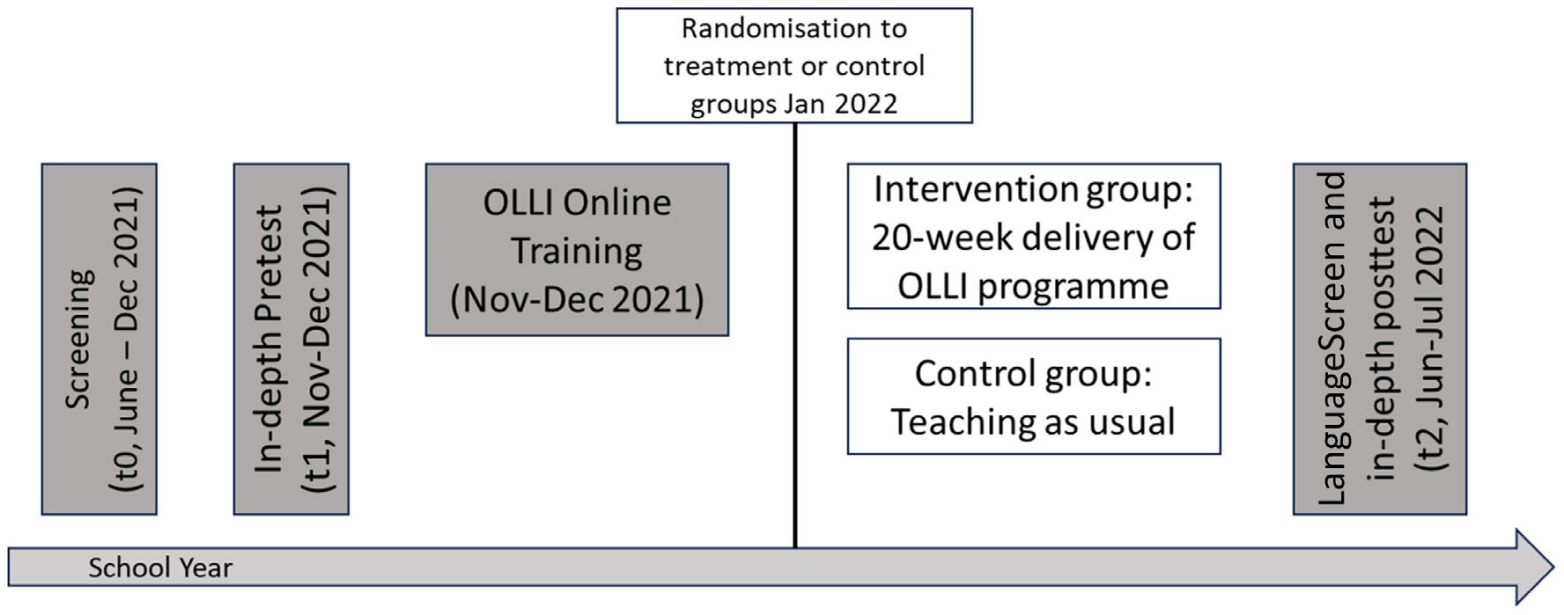
Timeline of the randomised controlled trial.

**Figure 2:**
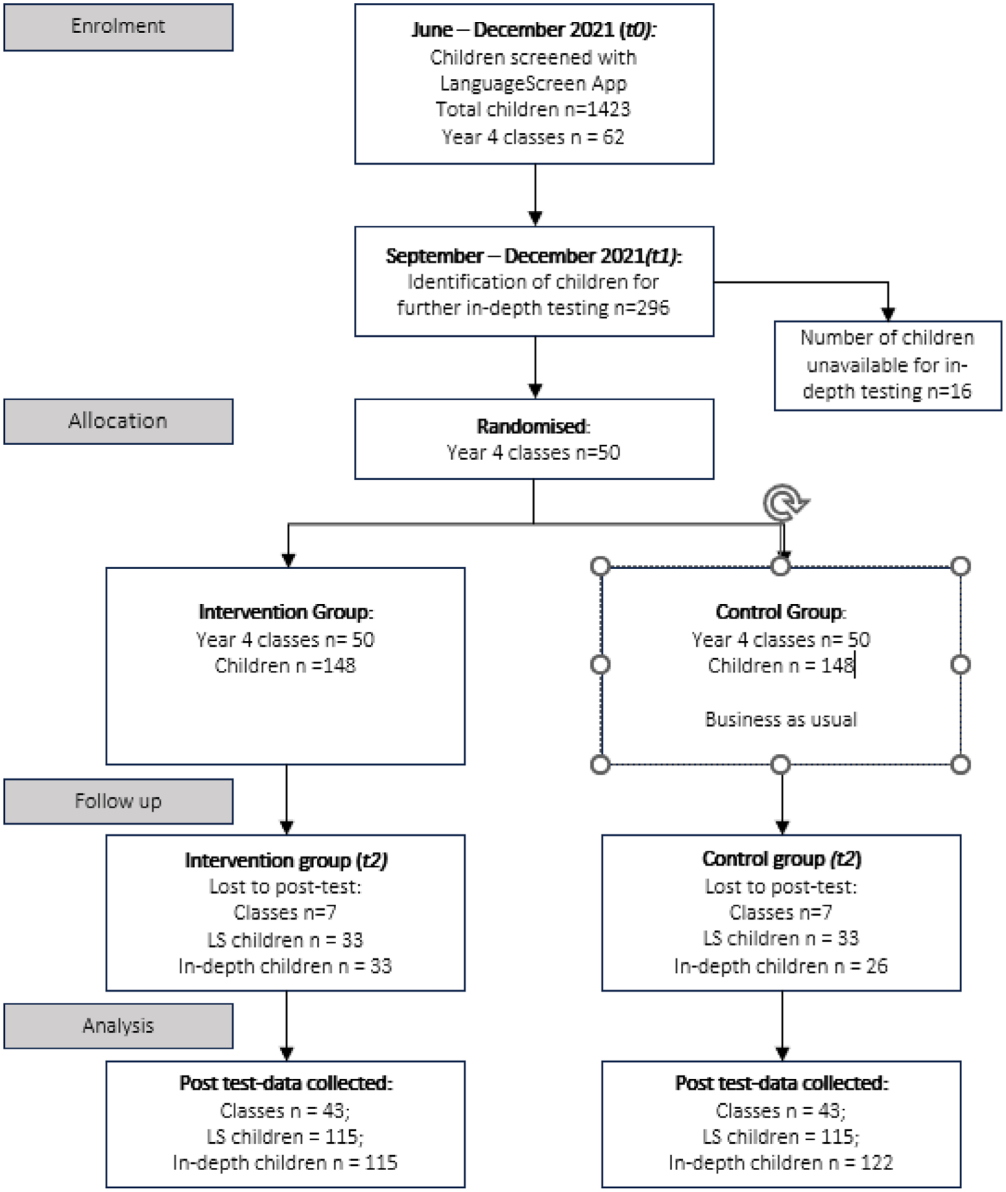
Flow of participants in the randomised controlled trial.

Following completion of the OLLI programme, all 6 children in each participating class were assessed again using LanguageScreen and the same battery of language, oral narrative and arithmetic measures as used at t1. The timeline presented in Figure 1 shows the assessment, training and intervention phases. Figure 2 shows the flow of participants through the trial.

### Measures

#### Screening

All children in the trial were assessed with LanguageScreen at t0 and at t2 (https://oxedandassessment.com/languagescreen/). LanguageScreen is a language assessment administered on a tablet comprising four subtests: Expressive vocabulary (24 items) requires children to name pictures; Receptive vocabulary (23 items) requires them to match a word they hear to one of four pictures on the screen; Sentence repetition (14 items) requires children to repeat sentences that they hear verbatim and Listening comprehension (16 items) involves the child providing answers to literal and inferential questions about three short stories that they hear.

#### In‐depth language assessments

Children receiving in‐depth tests were also tested on three subtests of the Clinical Evaluation Language Fundamentals (CELF‐4) (Semel, Wig, & Secord, 2006). In the Recalling Sentences subtest, the child is asked to repeat a series of sentences that are spoken by the examiner. The sentences increase in length and difficulty as the test progresses. In the Formulating Sentences subtest, the child is presented with a series of picture cards and asked to describe the picture in a sentence that uses a target word provided. Understanding Spoken Paragraphs requires the child to listen to a spoken paragraph and respond to a series of questions assessing the main idea, details, sequencing, prediction and inference. Children also received the Renfrew Action Picture Test (Renfrew, 2020), in which they were presented with a series of 10 picture cards and asked to answer a short question about each picture. Answers are recorded verbatim and scored for information content and grammar.

#### Additional measures

Children heard 12 ‘specifically targeted’ vocabulary items, which were directly taught in the intervention and six ‘not specifically targeted’ vocabulary items which, although not explicitly taught, did occur in the passages read aloud to them in the intervention. Children were encouraged to ask about the meanings of words they did not understand in the programme, so it is possible that they would have been given an explanation of the meanings of some of the not specifically targeted words. Children were asked to define each word.

Written narrative skills were assessed using the Writing Assessment Measure (WAM) (Dunsmuir et al., 2015). The children were presented with a verbal prompt, which was also printed on their test papers. The children were asked to write independently for 15 min. The written narrative produced was then scored on four criteria: sentence structure and grammar, vocabulary, organisation and overall structure and ideas. The total number of words produced was also recorded.

Finally, as a control measure, all 6 children in each class completed a 2 min arithmetic test from the Test of Basic Arithmetic and Numeracy (TOBANS; Brigstocke, Moll, & Hulme, 2016). The children were asked to complete as many addition or subtraction sums as possible in 1 min. The writing and maths tests were administered in groups by school staff.

### The OLLI Programme

The Oral Language for Literacy (OLLI) Programme is a targeted 20‐week manualised intervention for children aged 8–10 years. The programme is designed to improve children's oral and written narrative skills and enrich vocabulary knowledge and its application to spoken and written language. The programme promotes active listening and develops children's understanding of figurative language through jokes and idioms. Children are encouraged to develop metacognitive strategies to support comprehension of spoken language. Understanding language and oral narrative is also supported by the teaching and modelling of the use of reciprocal teaching (Palinscar & Brown, 1984).

The scripted intervention is delivered by trained teaching assistants. There are three 30 min sessions per week; two delivered to groups of three children and one individual session delivered to each child (see Appendix A1 for an example of a scripted session with accompanying resources). Each daily oral narrative introduces a new Tier 2 vocabulary item, the ‘word of the day’. Integrated into all oral passages are a range of other Tier 2 words that are not explicitly taught.

### OLLI teaching assistant and senior leader support

All teaching assistants selected by their schools to deliver the OLLI programme were required to attend live online training sessions alongside a senior leader from their school. The attendance of the senior leader was required to ensure that schools were provided with a clear overview of the programme and an understanding of the staff time required for effective delivery. The training stressed the importance of delivering the programme with fidelity and avoiding contamination from the intervention children to the control children (those who would have been eligible to receive the intervention but were not assigned to receive it).

The training was delivered over 2 half‐day sessions, beginning with an introduction to children's oral language development. The training then focused on the theoretical background of the programme, followed by detailed instructions covering the programme materials, session structure, activities and resources. Support and guidance were provided for school staff throughout programme delivery. Schools could access support through online meetings or telephone calls or could request a visit from the team to discuss any issues with programme or request a modelled session or an observation with feedback from the research team. Schools were contacted every 3–4 weeks throughout the intervention delivery period to check on progress and to offer support as needed.

## Results

All analyses were performed on an intention‐to‐treat basis. The majority of the analyses were conducted in Stata 18.0 (Stata Corp, College Station, Texas, USA). Structural equation models (SEM) were constructed using Mplus 8.10 (Muthen & Muthen, 1998–2023) with Full Information Maximum Likelihood estimators to allow for missing data. The analyses followed the preregistered plan (https://www.isrctn.com/ISRCTN78621766).

At screening (t0), 1423 children were screened with LanguageScreen. A total of 296 children were identified, based on their LanguageScreen scores, as eligible to receive intervention, however, 2 classes from a single school withdrew from the trial after in‐depth testing had been completed (children in this school were retained in the Intention to Treat analyses reported below). Children were then randomly allocated within schools to receive, or not receive, the intervention (148 children per group). At posttest, 33 control children (22%) and 33 intervention children (22%), from 7 classrooms, were lost to follow up. Critically, there were no significant differences at pretest in gender χ^2^ (1) = 0.05; *p* = .83, age (*d* = −0.19; 95% CI (−0.67, 0.19)) or LanguageScreen total scores (*d* = −0.04, 95% CI (−0.52, 0.44)) between children who completed the study and those who dropped out at posttest, indicating that attrition is unlikely to bias the estimates of effect sizes reported below.

Descriptive statistics for all measures at pretest and posttest for both the intervention and control group are shown in Table 1. The groups are well equated on language skills at the pretest.

**Table 1:** Mean raw scores (*SD*) for the intervention and control groups for primary and secondary outcome measures at screening (t0) preintervention (t1) and postintervention (t2), with effect sizes for intervention effects

	Reliability	*N*	Intervention		*N*	Control group		Cohen's *d*
			*n* = 148			*n* = 148		
			*M*	*SD*		*M*	*SD*	
Age (months)								
t0			99.70	4.12		100.23	3.80	
Language screen (t0 and t2)								
Expressive vocabulary								
t0‐(24)	.84	148	14.53	3.69	148	14.38	3.27	0.27^a^
t2‐(24)		115	17.56	3.59	115	16.73	3.30	
Receptive vocabulary								
t0‐RV (23)	.77	148	18.70	3.61	148	18.86	3.85	0.04^a^
t2‐RV (23)		115	22.55	1.93	115	20.43	2.06	
Sentence repetition								
t0‐SR (14)	.87	148	10.64	2.94	148	10.36	2.85	0.07^a^
t2‐SR (14)		115	11.71	2.83	115	11.40	2.38	
Listening comprehension								
t0‐LC (16)	.83	148	10.59	2.37	585	10.62	2.66	0.38^a^
t2‐LC (16)		115	12.63	2.37	115	11.63	2.59	
In‐depth tests (t1 and t2)								
CELF‐recalling sent								
t1‐(96)	.87	146	40.38	13.40	146	41.32	13.37	0.14^a^
t2‐(96)		115	48.68	13.67	122	48.28	13.93	
CELF‐formulating sent								
t1‐(48)	.84	146	27.14	9.49	146	27.08	9.91	0.24^a^
t2‐(48)		115	31.51	7.85	122	29.37	7.50	
CELF‐understanding para								
t1‐(15)	.82	8.09	8.09	2.91	146	8.23	3.16	0.34^a^
t2‐(15)		10.25	10.25	2.53	122	9.30	2.82	
APT information								
t1‐(40)	.86	146	28.11	5.08	146	28.70	4.50	0.38^a^
t2‐(40)		115	31.73	4.54	122	30.14	3.62	
APT grammar								
t1‐(36)	.74	146	22.74	5.26	146	22.95	5.10	0.31^a^
t2‐(36)		115	27.01	4.73	122	25.50	4.52	
WAM‐sentence struc								
t1‐(4)	.86	120	1.35	0.88	123	1.22	0.84	0.11^a^
t2‐(4)		98	1.78	0.84	98	1.56	0.92	
WAM‐organisation								
t1‐(4)	.85	120	1.08	0.64	123	1.11	0.73	0.40^a^
t2‐(4)		98	1.58	0.61	98	1.30	0.74	
WAM‐vocabulary								
t1‐(4)	.85	120	1.38	0.68	123	1.41	0.70	0.44^a^
t2‐(4)		98	1.95	0.85	98	1.65	0.79	
WAM‐ideas								
t1‐(4)	.84	120	1.20	0.77	123	1.19	0.78	0.42^a^
t2‐(4)		98	1.80	0.77	98	1.41	0.87	
TOBANS‐addition								
t1	.92	114	21.22	11.69	114	22.25	13.89	0.13^a^
t2		96	25.31	12.00	103	23.28	12.00	
Tobans‐subtraction								
t1	.88	114	16.80	9.61	112	16.68	11.98	0.01^a^
t2		96	19.40	10.37	103	18.65	13.23	
Bespoke vocab – targeted								
t1‐(12)	.94	146	2.05	2.22	145	2.21	2.48	1.06^a^
t2‐(12)		115	7.03	3.13	122	4.65	2.64	
Bespoke vocab – not targeted								
t1‐(6)	.94	146	1.30	1.14	145	1.29	1.39	0.35^a^
t2‐(6)		115	2.91	1.61	122	2.41	1.21	

^a^ Effect size for the intervention based on the difference in progress between groups from the ANCOVA model divided by pooled *SD* for the measure at t1 (see Morris, 2008); figures in brackets are the maximum possible score on each measure at each time point.

### Effect size estimates for the preregistered outcomes

The effects of the intervention were estimated with ANCOVA models, where the relevant latent outcome variable at t2 was regressed on both the corresponding latent variable at t1 and a dummy variable indicating group membership (intervention = 1 and control = 0). Before estimating the effect sizes from the latent variable models, confirmatory factor analyses (CFAs) for the pretest and posttest latent variables were estimated to assess the adequacy of model fit and to establish whether metric or scalar invariance held for the latent variables across time. In each model, the residuals for the same latent variable indicators were correlated, when significant, between the two times of measurement to provide an adequate model fit. The effects of the intervention were measured by the y‐standardised regression coefficient for a group dummy variable. The effects of clustering within schools were accounted for using robust (Huber–White) cluster standard errors. For each ANCOVA model, we checked for equality of slopes between the covariate (pretest factor) and outcome by including a group by covariate interaction term. In all cases, these interactions were small and nonsignificant and were, therefore, not included in the models reported. This indicates that the effects of intervention for all measures did not vary as a function of initial levels of the covariate; that is, children responded equally well to the intervention irrespective of their starting levels.

**Figure 3:**
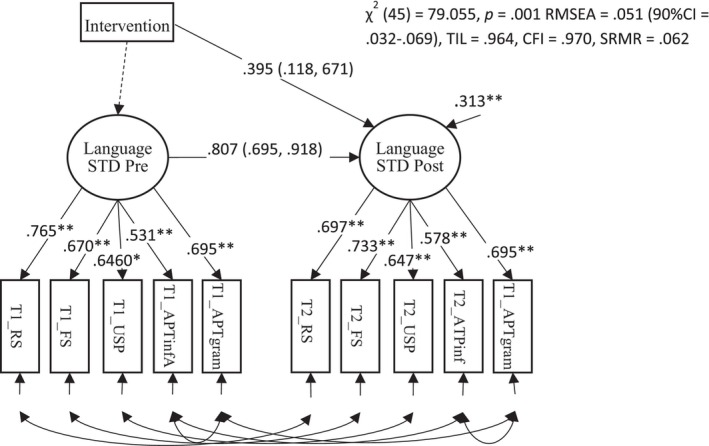
Path diagram for the pre‐reregistered primary outcome for the trial showing the effect of the intervention on standardised measures of language ability. The effect of the intervention is shown by the path from Intervention (dummy coded) to language at posttest, which is *y*‐standardised and equivalent to Cohen's *d*. 95% robust confidence intervals accounting for clustering within schools are shown in brackets ** p < .01

The pre‐registered primary outcome was a latent language variable created from the five individually administered language tests (CELF 4 Recalling Sentences; CELF 4 Formulating Sentences; CELF 4 Understanding Spoken Paragraphs and Renfrew Action Picture Test – Information and Grammar). An initial CFA model where only the correlations between the residuals of the same variable across time were estimated did not fit well. To improve the fit of the model the residuals for the two measures derived from the APT test (information and grammar) were correlated at each time point. This was deemed appropriate since these two scores are derived from the same responses given by the child. The resulting ANCOVA model is shown in Figure 3. The model fitted the data well and displayed scalar invariance (unstandardised factor loadings and intercepts did not differ across time (Wald test: χ^2^ (8) = 10.791, *p* = .214)). This model showed a substantial effect of intervention (*d* = 0.395, *p* < .001). (A preliminary model showed that the interaction between the pretest and intervention group (unstandardized β = .054, *p* = .668) was small and nonsignificant, indicating equal regression slopes in the two groups.)

**Figure 4:**
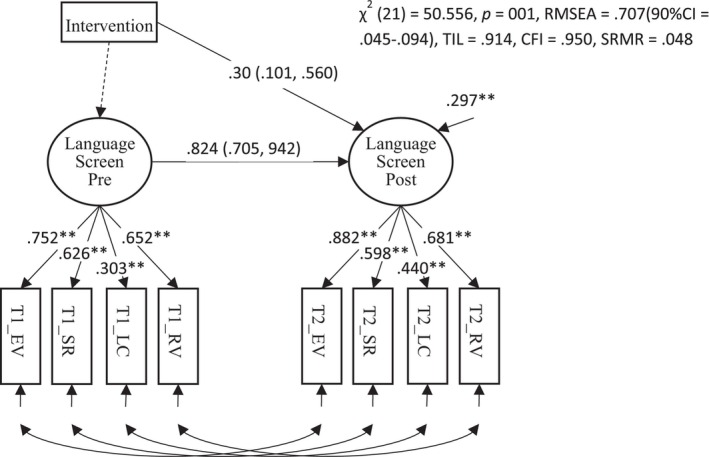
Path diagram for a pre‐reregistered secondary outcome for the trial showing the effect of the intervention on LanguageScreen. The effect of the intervention is shown by the path from Intervention (dummy coded) to language at posttest, which is y‐standardised and equivalent to Cohen's *d*. The 95% robust confidence intervals accounting for clustering within schools are shown in brackets ** p < .01

A secondary pre‐registered outcome measure was a latent language variable created from the four LanguageScreen subtests (Receptive Vocabulary, Expressive Vocabulary, Listening Comprehension and Sentence Repetition). This model is shown in Figure 4 and showed a mediocre fit to the data. The poor fit here appeared to reflect problems with the distribution of scores on the Receptive Vocabulary subtest; this measure was skewed due to a trend towards a ceiling effect. This model did not show metric invariance since the unstandardised factor loading varied between the two time points time (Wald test: χ^2^ (3) = 12.844, *p* = .005). The estimated effect size of the intervention from this model was substantial (*d* = .30, *p* < .001). (A preliminary model showed that the regression slopes did not differ between groups (unstandardized *β* = −.163, *p* = .449).)

**Figure 5:**
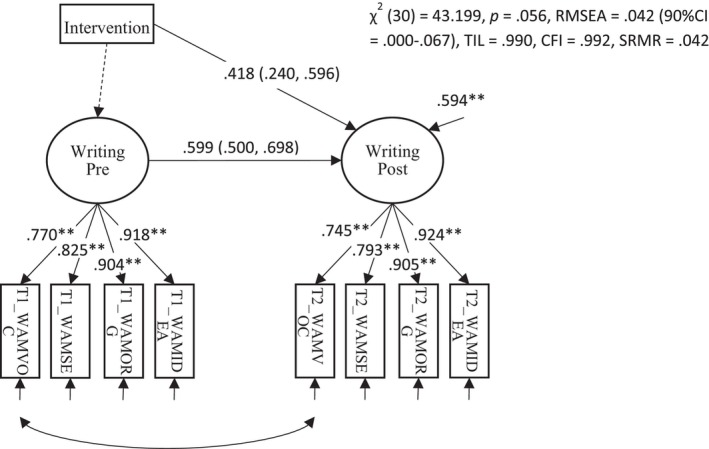
Path diagram for a pre‐reregistered secondary outcome for the trial showing the effect of the intervention on expressive writing skills. The effect of the intervention is shown by the path from Intervention (dummy coded) to language at posttest, which is y‐standardised and equivalent to Cohen's *d*. 95% robust confidence intervals accounting for clustering within schools shown in brackets ** p < .01

Another secondary pre‐registered outcome measure was a latent variable assessing children's expressive writing from the Writing Assessment Measure (WAM; Dunsmuir et al., 2015). This factor is defined by four measures derived from the WAM – sentence structure and grammar, organisation and planning, vocabulary and ideas. The model can be seen in Figure 5 and shows scalar invariance across time: Wald test: χ^2^ (6) = 7.465, *p* < .280. The estimated effect size of the intervention from this model was large and significant (*d* = 0.418, *p* < .001). (A preliminary model showed that the regression slopes did not differ between groups did not differ (unstandardized β = −.017, *p* = 0.902).) The effects here on the WAM show that an intervention that focuses purely on children's receptive and expressive language skills transfers directly to improvements in children's written expression, which is a key educational outcome for children of this age and above.

A further secondary pre‐registered outcome measure was a latent variable assessing arithmetic skills based on the 1 min addition and subtraction tests from the Test of Basic Arithmetic and Numeracy Skills (TOBANS; Brigstocke et al., 2016). This was a control measure since our oral language intervention was not expected to improve children's arithmetic skills. The model is shown in Figure S1 and showed scalar invariance (the unstandardized factor loadings and intercepts did not differ across time, Wald test: χ^2^ (2) = 2.916, *p* = .233). The estimated effect size of the intervention from this model was, as expected, trivial in size and not significant (*d* = 0.070, N.S.). (A preliminary model showed that the regression slopes were almost identical across groups, (unstandardized β = .029, *p* = .761).)

A final pair of secondary pre‐registered outcome analyses assessed whether bespoke specifically targeted vocabulary (i.e., defining words directly taught in the intervention) and bespoke not specifically targeted vocabulary (i.e., defining words of similar difficulty to the bespoke specifically targeted words, which were not specifically targeted in the intervention) were improved by the intervention. Hierarchical linear models with random intercepts for school, pretest as the covariate, posttest as the outcome and group (dummy coded) showed significantly larger improvements in scores in the intervention group on both specifically targeted (difference in marginal means = 2.56, 95% CI 1.97, 3.15; *d* = 1.09) and not specifically targeted vocabulary (difference in marginal means = 0.45, 95% CI 0.13, 0.76; *d* = 0.35). (In both cases preliminary models showed that the slopes relating outcome to pretest scores did not differ.) As expected, the improvements on the specifically targeted vocabulary are very large, but the effects on not specifically targeted vocabulary are also substantial in size.

#### Intervention dosage

Schools reported that on average the OLLI programme was delivered for 13.64 weeks out of a recommended 20 weeks (range 0–20); schools did not record dosage for individual children; 40% of schools delivered the programme for 10 weeks or less, 30% for between 13 and 16 weeks and 30% for between 16 and 20 weeks. Data from all schools were included in the analyses reported above, meaning that the effect sizes obtained may tend to be underestimates of the effect sizes expected if all 20 weeks of the programme were delivered.

## Discussion

Language difficulties are common in children in the late primary school years but, arguably, are rarely identified or addressed by schools. The OLLI programme evaluated here is a Tier 2 (Catts, 2009) intervention designed to improve the language and literacy skills of 8– to 11‐year‐old children who struggle with these skills. Language skills are closely intertwined with the development of literacy skills in this age range (particularly reading for meaning and written expression). The OLLI programme directly targets oral language weaknesses with the aim of improving both language and literacy skills, since oral language skills are critical for the development of both reading comprehension (Clarke et al., 2010) and written composition (Graham, 2019). The programme incorporates some ideas used in earlier related work (the Nuffield Early Language Intervention (NELI) Fricke, Bowyer‐Crane, Snowling, & Hulme, 2018) since there is an emphasis on the direct teaching of vocabulary and work on narrative production and comprehension skills. But, in addition, it incorporates work on more advanced aspects of language, particularly the understanding of nonliteral or figurative language, including work on metaphors, similes, humour and poetry (as developed in the Oral Language programme of Clarke et al., 2010).

The OLLI programme as evaluated here, produces substantial, and educationally meaningful (Kraft, 2020) improvements on standardised measures of oral language ability (*d*'s = 0.40 and 0.30) and children's written composition (*d* = 0.41). The programme also produces substantial improvements in specifically targeted vocabulary (*d* = 1.09) as well as improvements in knowledge of vocabulary not specifically targeted (*d* = 0.35) (cf. Clarke et al., 2010).

This study adds to the growing evidence that oral language interventions can be delivered successfully in school and produce meaningful improvements in language and literacy skills (Hulme, Snowling, West, Lervåg, & Melby‐Lervåg, 2020; West et al., 2021, 2024). Most of this research to date, however, has focused on younger children (preschool and early years). This study demonstrates that intervention in the late primary school years can be effective and provides materials that will allow schools to address these problems. Although this study, and others described above, show that language skills are to some degree malleable, they nevertheless appear to reflect a highly stable trait. We believe that many children will potentially benefit from structured input to facilitate their language development throughout an extended period of their education, ideally starting in the early years and continuing for several years in primary school.

### Limitations

The improvements in oral language and written composition found here are clear. It is unfortunate that, due to funding constraints, this study did not include measures of reading accuracy and reading comprehension. Based on earlier evidence (Clarke et al., 2010; Fricke et al., 2013) improvements in language comprehension might well transfer to improvements in reading comprehension, but not reading accuracy. This is an important issue to address in future trials of the OLLI programme.

The OLLI programme was developed as a Tier 2 programme and the data from this trial show it is effective. Feedback from schools indicates that the programme was well received by both school staff and pupils. Future work may usefully try to extend the ideas in this programme to a whole‐class programme so that all pupils could potentially benefit (cf. the NELI Preschool programme, West et al., 2024).

## Conclusions

This study demonstrates that the OLLI programme is effective in improving children's language and writing skills. The size of improvements is educationally meaningful, and suggests that the programme, given that it is scalable, and potentially of low cost, has important implications for educational policy. Language skills form a critical foundation for education. Language is the medium of instruction, it is critical for all aspects of literacy development, and is also important for psycho‐social development. Interventions to improve language skills are therefore important and should be given high importance in education. Interventions such as the one evaluated here may be particularly important for children from disadvantaged backgrounds and for immigrant children. At a broad level, reducing social inequalities in language, may transfer to improved educational outcomes, and thereby contribute to reducing social inequalities in educational outcomes and adult life chances.

## Trial registration

This study was preregistered: https://doi.org/10.1186/ISRCTN78621766.

**Key points**

- Oral language skills are critical for education and psychosocial development.
- Data from a randomised controlled trial show that a 20‐week language intervention delivered to 8–9‐year‐olds can produce educationally meaningful improvements in their oral language and writing skills.
- An automated app allows school staff to identify children with language difficulties to provide them with additional targeted language support.

## Supporting information


**Figure S1** Path diagram for a pre‐reregistered secondary outcome for the trial showing the effect of the intervention on arithmetic skills.

## Data Availability

Anonymised data for this study is available on request from the last author.
