# Supplementary material for: Oral language intervention in the late primary school years is effective: evidence from a randomised control trial
Source: J Child Psychol Psychiatry. 2024 Dec 1;66(6):775–84. doi: 10.1111/jcpp.14084 (PMC12062848; doi:10.1111/jcpp.14084)
Supplement: Supplementary file 1 — Figure S1 Path diagram for a pre‐reregistered secondary outcome for the trial showing the effect of the intervention on arithmetic skills. [file JCPP-66-775-s001.docx]

**Supporting Information:**

Additional supporting information may be found online in the Supporting Information section at the end of the article:

Figure S1. Path diagram for a pre-reregistered secondary outcome for the trial showing the effect of the intervention on arithmetic skills. The effect of the intervention is shown by the path from Intervention (dummy coded) to arithmetic skills at posttest, which is y-standardised and equivalent to Cohen’s *d*. 95% robust confidence intervals accounting for clustering within schools shown in brackets.

.923**

T1_Sub

.945**

T1_Add

.954**

T2_Sub

.903**

T2_Add

Intervention

.124**

χ^2^ (4) = 8.641, *p* = .071, RMSEA = .069 (90%CI = .000-.133), TIL = 991, CFI = .996, SRMR = .027

.935 (.887, .983)

.070 (-.082, .222)
